# Case Report: Adenosine-induced atrioventricular dissociation: unmasking monomorphic tachycardia as a diagnostic challenge in a neonate

**DOI:** 10.3389/fped.2025.1662114

**Published:** 2025-10-27

**Authors:** Feifei Wang, Bin Wu, Jiaqi Huang, Ba Yaletai, Peng Liu

**Affiliations:** ^1^ Department of Electrocardiogram Laboratory, Ordos Central Hospital, Ordos School of Clinical Medicine, Inner Mongolia Medical University, Inner Mongolia, China; ^2^ Department of Endocrinology, Qingdao Municipal Hospital, Qingdao, China; ^3^ Department of Cardiology, Ordos Central Hospital, Ordos School of Clinical Medicine, Inner Mongolia Medical University, Inner Mongolia, China; ^4^ Department of Neonatology, Ordos Central Hospital, Ordos School of Clinical Medicine, Inner Mongolia Medical University, Inner Mongolia, China

**Keywords:** monomorphic, tachycardia, neonate, electrocardiography, adenosine

## Abstract

**Background:**

Neonatal monomorphic tachycardia poses a diagnostic challenge. This report demonstrates how adenosine-induced AV dissociation confirmed ventricular tachycardia.

**Case:**

A 3-day-old preterm neonate (34 + 2 weeks) presented with refractory monomorphic tachycardia (217 bpm; QRS 92 ms) initially diagnosed as SVT based on 1:1 retrograde P-waves. Adenosine administration induced atrioventricular dissociation without termination—a finding inconsistent with SVT. Retrospective ECG analysis revealed prolonged QRS duration during tachycardia (92 ms vs. 60 ms post-cardioversion) and delta wave-like slurring, confirming VT diagnosis. Synchronized cardioversion (0.5 J/kg) restored sinus rhythm, followed by metoprolol prophylaxis.

**Conclusion:**

This case highlights that monomorphic tachycardia in neonates may represent VT. Adenosine's role in inducing AV dissociation is pivotal for diagnosis, and low-energy cardioversion with β-blocker maintenance offers an effective rescue strategy. Clinicians must reassess ECG features dynamically to avoid misclassification.

## Introduction

Neonatal arrhythmias, though relatively uncommon with an estimated incidence of 1% in general populations and up to 5% in neonatal intensive care units (NICUs), represent critical clinical challenges due to their potential for hemodynamic compromise and long-term sequelae (1). While most cases are transient and benign, non-benign arrhythmias such as supraventricular tachycardia (SVT) or ventricular tachycardia (VT) require urgent intervention to prevent cardiac failure or sudden death (2). This case report describes a 3-day-old preterm neonate with life-threatening relatively narrow QRS complex tachycardia tachycardia unresponsive to adenosine, successfully managed with electrical cardioversion and sustained by metoprolol.

## Case presentation

A male neonate was electively delivered via lower segment cesarean section at 34 + 2 weeks gestation to a 30-year-old primigravida with an unremarkable antenatal history. Serial fetal surveillance ultrasonography demonstrated normal anatomical development, complemented by negative maternal TORCH serological profiling. The procedure was performed under spinal anesthesia for persistent breech presentation, achieving Apgar scores of 9 and 10 at 1 and 5 min respectively. Neonatal biometric parameters included a birth weight of 2,180 g (30th percentile for gestational age).

**Figure 1:**
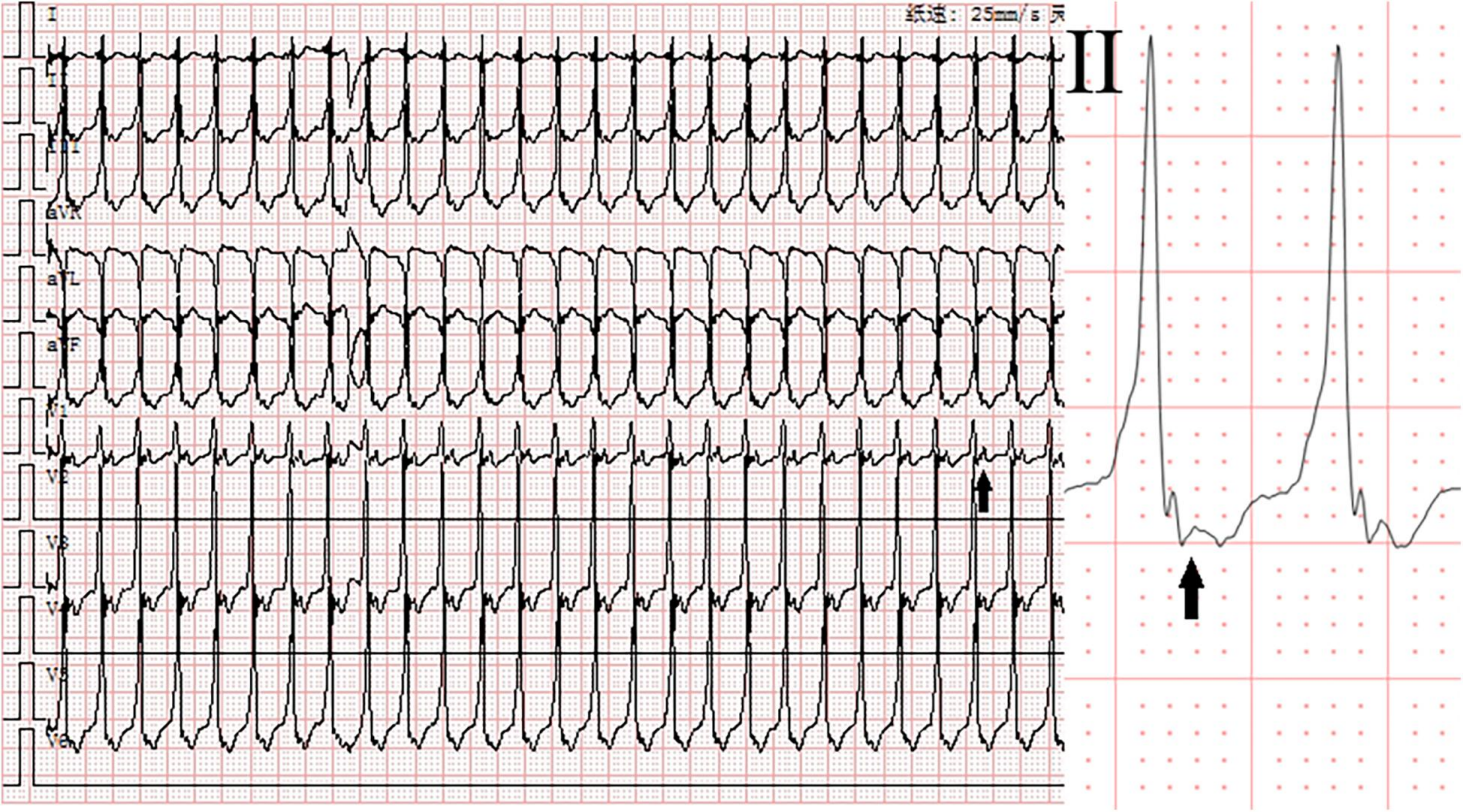
Electrocardiogram before adenosine. Monomorphic tachycardia (heart rate: 217 beats per minute, QRS duration: 92 ms) with retrograde P-wave (1:1). Solid arrows indicate P-waves.

**Figure 2:**
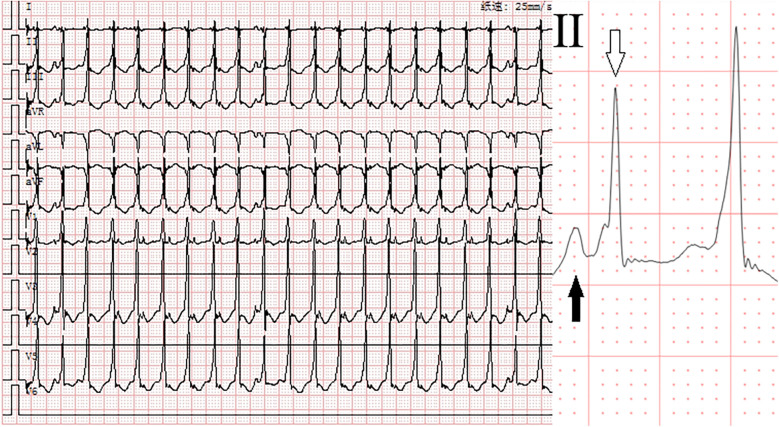
Electrocardiogram after adenosine. Monomorphic complex tachycardia (heart rate: 175 beats per minute, QRS duration: 92 ms) with atrioventricular dissociation and fusion beats. Solid arrows indicate P-waves, and hollow arrows indicate fusion beats.

**Figure 3:**
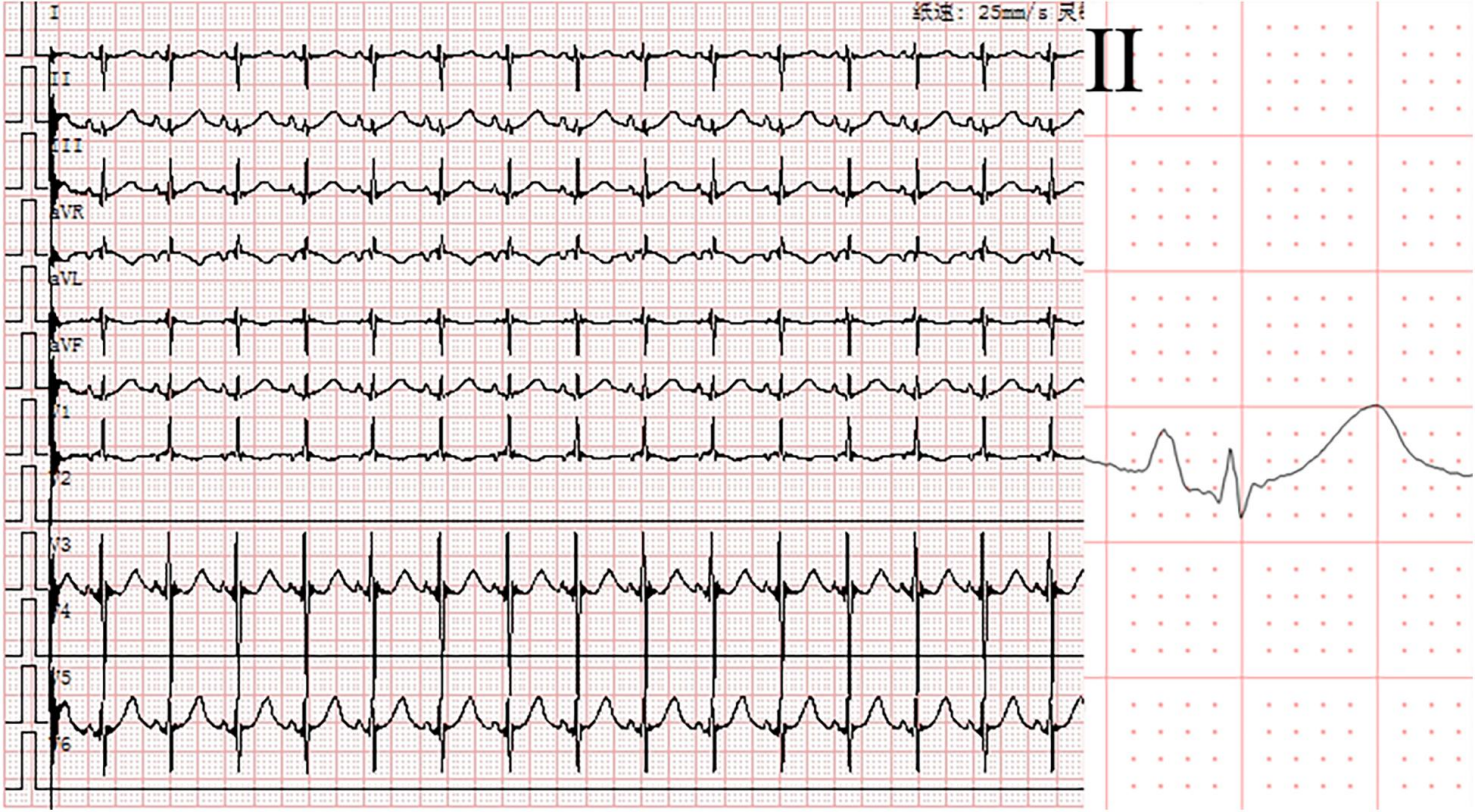
Electrocardiogram after electrical cardioversion. Sinus rhythm (heart rate: 123 beats per minute, QRS duration: 60 ms).

At 30 min postnatally, the infant developed acute respiratory decompensation manifesting as tachypnea (40 breaths/min), intercostal and subxiphoid retractions, with concurrent cyanosis. Radiographic evaluation identified decreased permeability of both lungs on chest x-ray. Echocardiographic evaluation confirmed transitional circulation patterns (patent foramen ovale with left-to-right shunting, patent ductus arteriosus) without structural anomalies. Laboratory analysis demonstrated marked elevation of cardiac biomarkers: White blood cell (WBC) 6.99 × 10^9^/L, Hemoglobin (HGB) 178 g/L. High-sensitivity troponin T (hs-TnT) 0.0605 µg/L (reference <0.014 µg/L) and NT-proBNP 3,596 pg/mL (reference <125 pg/mL). Hepatic derangement was evidenced by alanine aminotransferase (ALT) 15 U/L (13–35) and aspartate aminotransferase (AST) 37 U/L (7–40). Electrolyte profiling showed venous potassium 4.37 mmol/L (3.5–5.3) and magnesium 0.86 mmol/L (0.75–1.02), excluding significant ionic disturbances. Pulse oximetry demonstrated profound desaturation (SpO_2_ 84% in room air), prompting immediate escalation to non-invasive positive pressure ventilation (NIPPV mode: FiO_2_ 30%, PIP 16 cmH_2_O, Ti 0.35 s, RR 40 breaths/min). This intervention achieved partial oxygenation improvement (SpO_2_ 93%, pH 7.48, pO_2_ 82 mmHg, pCO_2_ 24 mmHg, Base Excess −5.6 mmol/L, and Lactate 2.2 mmol/L), while telemetry detected sustained wide-complex tachycardia (ventricular rate 217 bpm; QRS 92 ms) with a delta wave-like slurring at its onset and 1:1 conduction retrograde P-wave morphology, consistent with paroxysmal supraventricular tachycardia (Figure 1). Blood pressure (BP) was 68/35 mmHg. Intravenous adenosine administration (0.1 mg/kg) can reduce the heart rate to 175 beats per minute and show the phenomenon of atrioventricular dissociation and fusion beats with qrs-wave morphology unchanged (Figure 2). Ultimately, we diagnosed this tachycardia as ventricular tachycardia. Electrical cardioversion (0.5 J/kg) subsequently achieved successful sinus rhythm restoration (Figure 3) and BP was 70/34 mmHg. The infant's acute respiratory decompensation and profound desaturation were initially attributed to respiratory distress syndrome; however, their temporal coincidence with the onset of sustained tachycardia and the marked, immediate clinical improvement post-cardioversion strongly implicated the arrhythmia-induced hemodynamic compromise as the primary etiology. Initiation of metoprolol tartrate (0.1 mg/kg q12h) maintained arrhythmia-free status throughout 48 h monitoring and the patient maintained clinical stability with preserved cardiac function at 8-week reassessment.

## Discussion

Neonatal arrhythmias, particularly in preterm infants with structurally normal hearts, often pose diagnostic challenges (3). The neonate presented with refractory relatively narrow QRS complex tachycardia. Initially, the tachycardia was presumed to be supraventricular in origin based on the relatively narrow QRS duration (92 ms) and 1:1 retrograde P-wave morphology, prompting adenosine administration for attempted termination. Although adenosine failed to terminate the tachycardia, it played a crucial role in clarifying the diagnosis by inducing atrioventricular dissociation—a phenomenon inconsistent with SVT. Ultimately, the arrhythmia was successfully terminated via synchronized cardioversion. Additionally, retrospective comparison of the ECGs before and after cardioversion revealed two key features supporting ventricular tachycardia: (1) prolonged QRS complex duration during tachycardia (92 ms vs. 60 ms in sinus rhythm), and (2) delta wave-like slurring at the QRS onset. This diagnostic evolution underscores the importance of dynamic ECG reassessment and targeted therapeutic interventions in neonatal arrhythmia management.

The absence of structural heart defects or familial arrhythmia history in this case underscores the multifactorial nature of neonatal tachycardia (2). While congenital heart disease and electrolyte imbalances are common triggers, this neonate exhibited unremarkable echocardiographic and metabolic profiles. Elevated cardiac biomarkers (hs-TnT and NT-proBNP) further reflect myocardial strain, a phenomenon documented in arrhythmia-associated injury. We focus on supraventricular tachycardia (SVT) with aberrant conduction vs. ventricular tachycardia (VT). In this context, the administration of adenosine was instrumental. The induction of atrioventricular dissociation and fusion complex without termination of the tachycardia provided incontrovertible evidence for a diagnosis of VT, thereby excluding SVT with aberrancy as the underlying mechanism.

**Figure 4:**
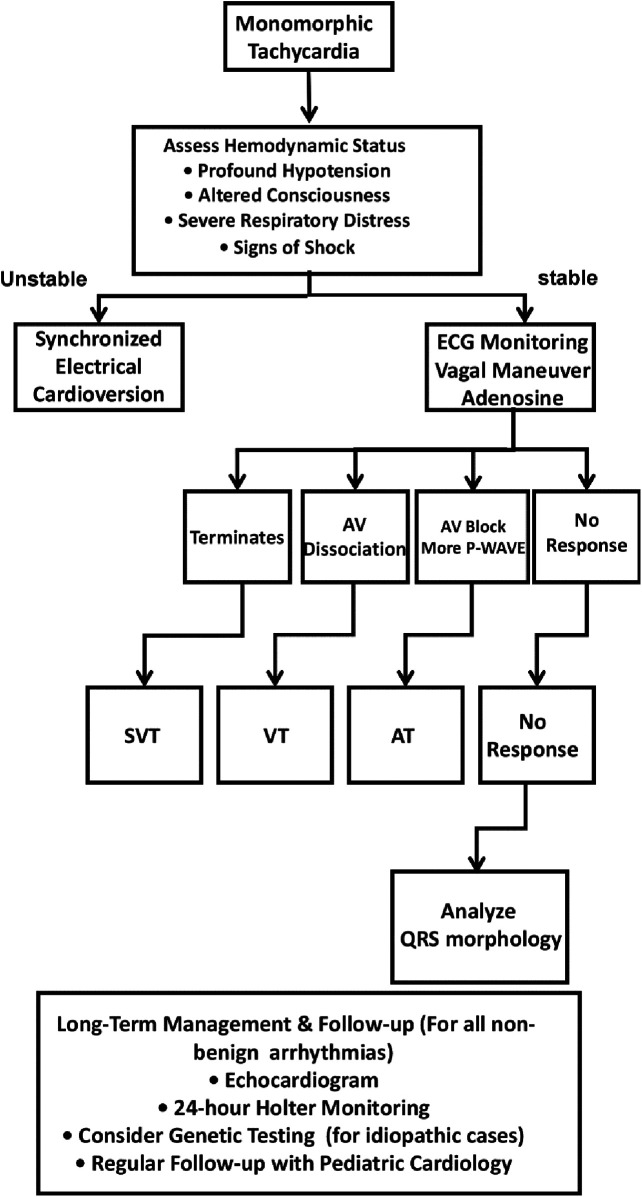
Proposed diagnostic algorithm for monomorphic tachycardia in neonates and infants. SVT, Supraventricular Tachycardia; VT, Ventricular Tachycardia; AT, Atrial Tachycardia.

We wish to underscore that the administration of adenosine (or the use of vagal maneuvers) during continuous 12-lead ECG monitoring is a critical diagnostic step in the evaluation of every monomorphic, relatively narrow-complex tachycardia. This approach is essential not only for potential termination of SVT but, as demonstrated in this case, for its ability to induce atrioventricular dissociation—a pathognomonic sign of VT. Furthermore, a meticulous analysis of QRS morphology, including the presence of slurring or patterns suggestive of underlying cardiomyopathy, should be routinely performed alongside response to adenosine. Based on the diagnostic challenge encountered in this case, we propose a systematic diagnostic algorithm for clinicians facing regular tachycardia in neonates (Figure 4).

Electrical cardioversion—a strategy supported by recent guidelines for hemodynamically unstable arrhythmias. The successful restoration of sinus rhythm at 0.5 J/kg aligns with recommended energy doses (5–15 J), minimizing myocardial injury risk. Post-conversion metoprolol maintenance prevented recurrence. In this case, a comprehensive echocardiogram ruled out structural anomalies such as cardiac tumors or cardiomyopathy. In the context of idiopathic VT with a structurally normal heart, as was present here, empirical beta-blocker therapy is a standard and often effective prophylactic strategy to suppress adrenergically-mediated triggers. The patient's sustained positive response to metoprolol supports this approach, though long-term follow-up and consideration of advanced genetic testing remain important.

The patient's sustained stability at 8 weeks supports the efficacy of combined electrical and pharmacological intervention. However, neonatal arrhythmias even when transient may signal latent conduction abnormalities, warranting extended follow-up. Studies indicate that 13.4%–25% of neonates with SVT/VT exhibit recurrence within the first year, often associated with accessory pathways or channelopathies (4, 5). Genetic testing was not performed here but should be considered in idiopathic cases to exclude inherited arrhythmogenic disorders.

## Conclusion

This case report underscores that life-threatening neonatal arrhythmias, including monomorphic tachycardias, may manifest in the absence of structural cardiac anomalies or familial predispositions, thereby presenting formidable diagnostic and therapeutic challenges. The diagnostic intricacy is accentuated by the pivotal role of pharmacologic agents that induce atrioventricular block, which can facilitate the differentiation of the underlying arrhythmogenic mechanisms. Expedient intervention, encompassing synchronized cardioversion when indicated, in conjunction with β-blocker prophylaxis, constitutes a salutary and efficacious management paradigm, particularly in preterm neonates undergoing transitional circulatory adaptations.

## Data Availability

The original contributions presented in the study are included in the article/Supplementary Material, further inquiries can be directed to the corresponding author.
